# An interpretative phenomenological analysis of the experience of a nature-based therapy intervention for children with long-term health conditions and associated psychological difficulties

**DOI:** 10.1177/13591053251315380

**Published:** 2025-02-07

**Authors:** Farhin Bhatti, Tamara Leeuwerik, Charlotte Savins, Lana Jackson

**Affiliations:** 1 Canterbury Christ Church University, UK; 2 St Ann’s Hospital, UK; 3 Worthing Hospital, UK

**Keywords:** children and young people, long-term conditions (LTC), mental health, nature-based therapeutic interventions, wellbeing

## Abstract

Children and young people (CYP) with long-term health conditions (LTC) are at higher risk of developing mental health difficulties. Research suggests nature-based therapeutic interventions (NBTIs) may benefit CYP’s wellbeing, but less is known about the impact on CYP with LTC. This study’s objective was to explore how CYP with LTC and associated psychological difficulties experienced a NBTI and the impact on their wellbeing. Ten participants aged 10–13 attended a NBTI and took part in semi-structured interviews that explored how they made sense of their journey through the intervention, its impact on mental, physical wellbeing and sense of self. An interpretative phenomenological analysis of the interview data yielded four group experiential themes: ‘Overcoming Illness-Identity’, ‘Freedom to Choose’, ‘Sense of Connection’ and ‘A Mindful Presence’. Participants reported improved self-esteem, a deepened sense of belonging with peers and nature, and enhanced emotion regulation. Clinical implications and directions for future research are discussed.

## Introduction

The biophilia hypothesis (Wilson, 1984) suggests humans have an innate tendency to connect with nature, enhancing internal wellness. Substantial research highlights both physiological and psychological benefits of nature exposure. Twohig-Bennett and Jones’ (2018) meta-analysis of 103 observational and 40 interventional studies showed significant reductions in blood pressure, salivary cortisol, diabetes, and cardiovascular mortality after time spent outdoors. Other studies found nature contact increases positive affect, self-compassion, and mindfulness, while reducing negative affect and impulsivity (Coventry et al., 2021; McMahan and Estes, 2015; Repke et al., 2018; Swami et al., 2019). The Stress Reduction Theory (Ulrich, 1981) may explain these restorative effects of nature, suggesting nature exposure facilitates psychological and physiological processes that ease stress and can speed up recovery from physical illness (Ulrich, 1984). Moreover, Kaplan and Kaplan’s (1989) Attention Restoration Theory suggests that nature engagement allows ‘soft fascination’, aiding reflection and introspection, improving cognition and mental clarity.

Ecotherapy encompasses interventions involving nature. Nature-based therapeutic interventions (NBTIs) are aimed at improving wellbeing and occur in green spaces from city parks to nature reserves, often as structured group programs led by professionals. Types of NBTIs include wilderness therapy (Berman and Davis-Berman, 2013), horticulture programs (Kamioka et al., 2014), nature-based education (Mann et al., 2021), and mindfulness programs (Djernis et al., 2019).

Concerns about deteriorating wellbeing among children and young people (CYP) have increased interest in NBTIs’ benefits. In the UK, 1 in 6 individuals aged 5–16 had a mental health problem in 2020, up from 1 in 9 in 2017 (Peytrignet et al., 2022). Causes are complex, but growing evidence suggests COVID-19 and associated social isolation have impacted CYP’s mental health (Kauhanen et al., 2023; Loades et al., 2020). A meta-analysis of 29 studies showed higher rates of depression (1 in 4) and anxiety (1 in 5) among young people globally post-pandemic (Racine et al., 2021). CYP now spend less time outdoors and more time online, leading to concerns regarding the negative impact of their disengagement from the natural world (Larson et al., 2019). CYP’s wellbeing is integral to their psychosocial development, relationships, and identity formation (Erikson, 1968). Therefore, supporting wellbeing in early life can prevent persisting difficulties into adulthood (Costello, 2016).

Systematic reviews evaluating the impact of NBTIs on CYP’s wellbeing have found that actively engaging with the natural world is associated with improved self-esteem, nature connectedness, behavioural functioning and reduced stress and depression (Arola et al., 2022; Roberts et al., 2020; Tillmann et al., 2018; Zhang et al., 2020). However, the existing literature has tended to exclude CYP with long-term health conditions (LTC) who experience associated psychological difficulties.

Approximately 15% of CYP internationally have LTC (Van der Lee et al., 2007). In the UK, 1.7 million CYP live with LTC such as asthma, diabetes, and epilepsy (National Institute for Health and Care Excellence, 2019). CYP with LTC are four times more likely to experience social and emotional difficulties than their physically healthy peers, particularly anxiety, depression, low self-esteem, and loneliness (Maes et al., 2017; Moore et al., 2019). This may be due to additional biopsychosocial challenges like pain, restrictions on recreational activities, school absence, frequent hospital visits and unexpected medical procedures (Golden et al., 2008). The limitations imposed by a LTC can also increase feelings of disempowerment, making accessing psychological support feel further stigmatizing, and delay access to treatment (Lerwick, 2016).

While there is evidence that NBTIs are associated with post-intervention improvements in psychological wellbeing in adults with LTC with associated psychological difficulties (Taylor et al., 2022; Trøstrup et al., 2019), research on CYP is limited and mostly focuses on physical health (Lee et al., 2014). Results from a small number of qualitative studies that have explored the participant experience of outdoor summer camps for CYP with LTC suggest NBTIs may support children with LTC to experience freedom, creativity, body empowerment and relaxation. However, these studies mainly explored the experience of the intervention, emphasising peer relationships and skill building, rather than engagement with the natural world, and fail to explore the meaning children with LTC and APD may derive from being in nature (Desai et al., 2014; Gillard and Allsop, 2016; Moola et al., 2014). Van der Riet et al. (2017) conducted a narrative study exploring the perceived benefit of healing gardens on the wellbeing of CYP with LTC and found that the gardens provided a destigmatising environment where CYP could shed their illness identity and stimulate their imagination. However, the study had methodological limitations and solely obtained the views of staff facilitating the NBTI, excluding CYP perspectives.

NBTIs may be more effective than indoor therapy in increasing equity of care, accessibility and ownership (Cooley et al., 2022). However, professionals rarely incorporate NBTIs within treatment planning due to a lack of experience and organisational barriers (Cooley et al., 2020; Wilkinson et al., 2019). A systematic review by Moore et al. (2019) evaluating psychological intervention for CYP with LTC and APD found interventions were most effective when offering a safe space, freedom, self-esteem, hope and social connection. This suggests that NBTIs may be well-placed to facilitate this as they offer CYP an opportunity to experience new challenges, alongside peer support, which can increase self-confidence and development of a positive sense of self (Duerden et al., 2012). This is vital for CYP with LTC, who often have illness-defined self-representations (Law et al., 2014) and limited nature access, increasing psychological distress vulnerability (Jimenez et al., 2021). NBTIs could support the healthy psychosocial development of CYP with LTC and APD (Erikson, 1958). Moreover, NBTIs offer safer socially distanced interventions for CYP with health vulnerabilities (Cooley and Robertson, 2020).

Exploring the lived experience of NBTIs for CYP with LTC and APD will contribute to understanding whether theoretical frameworks on the restorative effects of nature (Kaplan and Kaplan, 1989; Wilson, 1984) extend to this population and their unique biopsychosocial challenges. Within clinical practice, understanding how NBTIs serve as therapeutic alternatives for CYP who face stigmatization and difficulties engaging with traditional indoor therapies may encourage more widespread integration of NBTIs in the treatment of CYP with LTC and APD by mental health practitioners. Given the pressing concerns regarding the mental health crisis among CYP (Kauhanen et al., 2023) and the increased vulnerability within CYP with LTC (Moore et al., 2019), further exploration of the impact of NBTIs for this population may have helpful policy implications, for example, promoting the integration of NBTIs into educational, public health and social care strategies for CYP with LTC, particularly within vulnerable populations with limited access to nature.

The current study aimed to develop an understanding of the lived experience of CYP with LTC and APD participating in a NBTI designed to support wellbeing. The study explored the research question: How do CYP with LTC and APD experience the NBTI and how do they perceive the impact of the intervention, if any, on their wellbeing and sense of self?

## Methods

### Design

The study involved the interpretative phenomenological analysis (IPA) of individual semi-structured interviews with 10 CYP with LTC and APD who attended a NBTI. IPA is concerned with exploring how people make sense of, relate to, and perceive subjective experiences and often transformative events (Smith et al., 2021). This approach was therefore well-suited to address the research aims.

### Intervention

Participants took part in an NBTI within a woodland nature reserve. This one-day woodland intervention (WI), facilitated several times a year by a paediatric psychology service in a UK NHS hospital, is with groups of 8–10 children aged 9–13 years of age and involves mindfulness and forest school activities like fire lighting, nature arts and crafts, sensory games, and cooking.

### Recruitment and participants

Purposive sampling was used to recruit participants from the paediatric psychology service of a UK NHS hospital. CYP referred to the service have a range of LTCs alongside psychological difficulties, such as low mood and anxiety. Clinicians introduced the study to eligible children’s parents/carers (see Table 1 for the eligibility criteria), who received information sheets and a video. Interested parents/carers gave consent for contact details to be shared with the lead researcher. Two potential participants declined participation due to interview anxiety or time constraints. Participants completed electronic consent (parents) and assent (CYP) forms before the interview.

**Table 1.** Eligibility criteria.

Inclusion	Exclusion
Aged 9–13 years old	At current high risk of self-harm or suicide, as per their most recent assessment in the psychology service of a paediatric department
A diagnosis of a long-term physical health condition	
Open to the psychology service within the paediatric department of the hospital.	
Attended at least one WI	

WI: Woodland intervention.

Ten participants were recruited to the study, in line with IPA guidance (Smith et al., 2021). Demographics are summarised at group level to preserve participant anonymity (Table 2). LTCs included asthma (*n* = 2) coeliac disease (*n* = 1), neurofibromatosis-1 (*n* = 1), cancer (*n* = 1), bronchiectasis (*n* = 1), respiratory and liver disease (*n* = 1), immunodeficiency disorder (*n* = 1), epilepsy (*n* = 1), foetal alcohol spectrum disorder (*n* = 1).

**Table 2.** Participant demographic information.

Demographic categories	Group level statistics
Age	M = 12.2, SD = 2.6, Range = 10–13
Gender	F = 80%, M = 20%
Ethnicity	White English = 70%, mixed White and Black African = 20%, other mixed background = 10%
Number of WI attended	Mo = 1, Mdn = 2, Range = 1–4

### Data collection

Demographics were collected at the start of the semi-structured interview, prior to audio recording. The semi-structured interview questions were developed in consultation with two CYP and one parent who were open to the service but not participating in the study. The questions explored experiences of the WI, focusing on thoughts, feelings, and perceptions (Smith et al., 2021). The interview schedule explored CYP’s hopes for the WI, experiences of the WI, changes they noticed, the sense they made of these changes and how they felt this impacted their lives.

All participants opted for online rather than in-person interviews. Participants either chose to attend on their own, or with a parent/carer, to support their sense of safety. Four CYP chose to have their parent present for the main interview, for the others a parent joined briefly before and after the interview. The interviews were audio-recorded and lasted 35–60 minutes.

The researcher provided a de-brief after each interview and could signpost to further support if requested. Interviews were transcribed and anonymised prior to analysis. Participants received a summary of the research findings.

### Data analysis

As IPA takes an idiographic approach, each interview transcript was analysed individually before producing group themes or statements, ensuring analysis was centred on the unique experiences of each participant (Smith et al., 2021). A critical realist approach was applied within this methodology and data analysis (Willig, 1999). The analysis proceeded through the following iterative steps; (1) data familiarisation by twice reading through the transcript, allowing initial thoughts to emerge, (2) creating initial, comprehensive and detailed exploratory notes (EN) in the margin of the transcript, protracting initial descriptions, analytic comments and linguistic interpretations and reflections, (3) deriving experiential statements (ES) by grouping and summarising EN into preliminary constructs capturing essential aspects of participant experiences, (4) ES grouping into connected themes called personal experiential themes (PET). Themes were checked against the transcript to ensure they reflected the participant’s account (steps 1–4 were repeated for each transcript) (5) PETs for all cases were compared, identifying similarities and differences. Themes were refined to encapsulate collective experiences while mindful of individual nuances. Therefore, themes were not based solely on frequency but significance and richness. Group experiential themes (GET) and subthemes were developed through comparative analysis and creating overarching concepts encapsulating the entire dataset. Finally, transcripts were reread to ensure theme validity and to select verbatim quotes to illustrate each theme.

### Quality assurance

The first author is a South Asian British female trainee clinical psychologist who conducted the study towards their professional qualification. She has experience of working with CYP in child and adolescent mental health services. LJ is a White British female clinical psychologist specialising in CYP mental health and paediatrics. CS is a White British female arts psychotherapist with additional training in forest school leadership and extensive experience in paediatric mental health. LJ and CS delivered the WI. TL is a White Dutch female research tutor on a clinical psychology training course and clinical psychologist with expertise in CYP mental health who was not involved in the delivery of the WI. The authors value engaging with nature and holistic approaches to therapy and paediatric psychology, so may have held inherent views about the benefits of this intervention. However, they wished to gain an understanding of how CYP with LTC and APD experience an NBTI and wanted to understand how personal meaning and change in wellbeing was attributed to this experience.

Quality assurance was achieved by employing the hermeneutic cycle of suspicion and empathy (Smith et al., 2021), questioning assumptions and biases in the data alongside attending to individual stories and meanings attributed to experiences. Researcher reflexivity was key in developing awareness of biases, assumptions and pre-conceptions influencing data interpretation. A bracketing interview (Rolls and Relf, 2006) was carried out before data collection to help the researcher acknowledge how personal interests and values may influence the interpretation of the data (e.g. personal interests in holistic approaches and previous experience working with children in psychological settings) as well as recognising potential power imbalances and areas of conflict that may influence data collection (e.g. being an adult interviewing children and social desirability bias). Potential influences on the research process were also discussed in supervision. A reflexive log was completed at regular stages within the research process (Smith, 2006). Theme development was discussed at regular intervals with the research supervisors (LJ and TL) and research consultant (CS) to authenticate the integrity of theme development (Alase, 2017).

### Ethics

Ethics approval was obtained from an NHS research ethics committee (North East – Tyne & Wear South Research Ethics Committee: 22/NE/0042). Parental/carer informed consent and child assent was obtained. Participants were made aware they could withdraw or modify their consent at any point. Participants were informed that the information and personal details shared would remain confidential and anonymised, unless a concern of risk of harm to any individual was raised. People with LTC may experience fatigue and feel burdened by a lengthy interview. Therefore, participants were offered opportunities to take a break or end the interview if they wished. Participants or parents/carers who experienced distress, would be signposted to relevant support.

## Results

Four GETs were developed from the interview data (see Table 3). The GETs and their associated subthemes are discussed below. Pseudonyms are used within this section to protect participant anonymity.

**Table 3.** Overview of group experiential themes and subthemes.

Group experiential themes	Subthemes
Challenging illness identity	Desires and fears for change
	Exceeding expectations
	Pride and accomplishment
Freedom to choose	A release from restriction
	Agency to feel safe
	Creative exploration
Sense of connection	A shared struggle
	Weaved into nature’s road
A mindful presence	Engaging the senses
	Focusing the mind

### Theme 1: Challenging illness identity

Many participants believed living with a LTC meant being unable to engage with certain activities or goals, due to experiencing fatigue or mobility issues, which impacted their sense of self-efficacy. This GET encapsulated the way participants felt the WI offered an opportunity to challenge perceived limitations relating to their illness. Participants were surprised by their achievements, and this shifted how they viewed and experienced their illness identity. Many described a sense of accomplishment and feeling hopeful about their future.

#### Desires and fears for change

Almost all participants felt that living with a LTC had impacted their ability to be active and spend any length of time outdoors. Participants expressed the WI presented an opportunity to connect with previously inaccessible values: ‘*I wanted to like get better, to be like more fit and just do more stuff. So, it was almost a relief to get out and do that kind of stuff*’ (Oliver).

The sense of hope the WI offered in relieving feelings of stuckness was prevalent in many participants’ accounts. For example, Amara felt the WI would allow her to take positive risks and connect her with her ability rather than her disability: ‘*Usually I make it so I*’*m in my comfort zone a lot, but I wanted to be able to push myself so I feel like I*’*m able to actually achieve something*’.

However, alongside their desires, many participants expressed an awareness of the potential challenges the WI may present, such as the length of the day or physically demanding activities: ‘*I was worried about what we*’*re going to do. I can get tired a lot, with hypermobility*’ (Summer).

Participants seemed to express cautiousness and anticipation of potential threats to their illness. For some, this raised self-doubt and negative self-talk, exacerbating their anxiety: ‘*Will I screw it up…*’ (Noah) However, for all participants it seemed the benefits of the NBTI outweighed their fears, allowing them to move through these feelings.

#### Exceeding expectations

There was a sense from participants that living with a LTC often meant having limited experiences and opportunities to challenge yourself. Therefore, a chance to participate in stimulating activities, felt like a welcome surprise:

Cos I guess before I wasn’t able to do much and just staying indoors a lot so it kind of surprised me that like, I would actually get to do like, things like that. It felt nice and was like, oh I actually can do things again. (Noah)

Noah described how restrictions from his LTC had impacted his sense of what he was able to do, and the WI had allowed him to perceive a greater sense of what he was capable of. This was shared by many participants including Rachel, who was surprised by the expectations facilitators placed on participants: ‘*Well, I really didn*’*t think they would expect us to do so much. I thought we were going to be like, all sat down talking about things we did…*’

For many participants, this novel experience of feeling encouraged after a period of not feeling able, was highly motivating and supported self-confidence. Rachel shared that although she had not expected to be able to light a fire, she had strived to work hard to complete the activity: ‘*I felt really happy and relieved because one, my back was really hurting and two I had never made one before, and it was like really surprising I did it*’. It appears by participating in a novel, physically demanding activity, Rachel could explore the boundaries of her body’s capability, feeling reassured in her ability.

#### Pride and accomplishment

All participants expressed a sense of gratification from participating in the WI. There was a shared belief the difficulty and perseverance required to complete the activities such as fire lighting was integral to feeling accomplished. Kaylee reflected this was a personal learning experience, developing resilience and shaping a more hopeful perspective in response to life’s challenges: ‘*It felt, like, really satisfying. It felt like I*’*ve done something. Like an important moment to remember I just need to keep trying things if I want to. Well, practice makes perfect, basically*’.

Values of perseverance were similarly explicit for other participants, who expressed accomplishment was not solely derived from the successful completion of the nature-based activities, but by ‘*trying your best*’ and overcoming personal obstacles. This feeling of validation was shared amongst participants, who described this as a visceral feeling: ‘*When you first lit the fire, it warmed you up physically and mentally because it was quite cold, it was also kind of nice to know you lit something*’ (Grace). Grace’s awareness of a physical and mental warmth suggested a felt sense of contentment within her body.

Through completing challenging activities, participants seemed to have developed a sense of pride in their own tenacity, which seemed important for them to cope with the daily adversity of illness. This resulted in participants shifting from self-doubt to increased sense of confidence and hope regarding their future: ‘*I felt like if I can do this, it makes me feel like wow, I can do much more now*’ (Noah).

### Theme 2: Freedom to choose

This GET represented how the WI as well as the natural environment empowered participants to freely explore and make choices. Participants often described having reduced opportunities to make decisions for themselves due to their LTC, therefore the WI provided a novel, valued opportunity to express choice.

#### A release from restriction

The participants reflected on the invitational nature of the WI, allowing them to take care of their needs, listen to their bodies and make personal choices, alongside an implicit sense of freedom within nature: ‘*To feel I can do what I want, and go where I want, when I want, to makes a big difference. Because I*’*ve had a lot of like, controlling things with my epilepsy*’ (Kaylee). Kaylee had described experiences of being closely monitored and restricted from activities to manage her seizures, therefore the choice to explore without limitation felt freeing. This distinction between cautiousness and freedom was echoed in other participants’ comparing nature to the restriction of hospital settings: ‘*Cos in hospital everything is usually like this is a risk or be careful about that thing. So, it felt like woah! I was not expecting that…*’ *(Grace).* Grace’s expressive language indicates the excitement and surprise to exercise her independence. This sense of freedom within nature was reported to have a positive healing impact on participants’ perceived physical health, permitting greater ownership over their bodies. Noah who attended the WI shortly after a lobectomy expressed feelings of expansiveness and a capacity to breathe freely in nature: ‘*Inside it*’*s like you just have this air but outside you get like lashes of air going through you- it just felt nice and open and like relief to be there*’.

#### Agency to feel safe

Many participants expressed the significance of having agency to navigate their safety within what they perceived, at times, to be an overwhelming environment. Oliver, who was diagnosed with cancer, shared feeling accustomed to time alone while recovering from surgery, therefore valued the choice to be apart from the group: ‘*Yeah it was nice to have the time alone sometimes. As I hadn*’*t really been around people in a while. It*’*s nice to have that option as well…*’

Being granted the flexibility to make intuitive choices to regulate one’s own emotions and feel secure, was meaningful for many participants, who struggled with anxiety. Alongside the flexibility in rules on the WI, some participants felt the openness of nature allowed them to have ownership over their environment: ‘*I think if it was indoors and we were in one space, or a room, I wouldn*’*t have been able to feel as calm*… ’ (Beth).

The harmony of safety and freedom was explicitly important for many participants to feel secure enough to challenge themselves. Lydia expressed her gratitude for fair rules coinciding with her goals: ‘*There were only a few rules and they were sensible rules*’. However, navigating this balance was tricky for all participants, with some feeling exposed and vulnerable to dangers towards their health, particularly around the campfire: ‘*I felt like it was too dangerous. It kept getting into my lungs and my eyes and I felt like it could have been a bit more safe*’ (Amara).

Other participants shared both feeling exhilarated by the sensory experience as well as fears of being overpowered by it: ‘*It felt exciting but it just left like, oh gosh she*’*s going to come out and burn us all down*’ (Grace). Some participants shared that focussing on certain sensory aspects of nature enabled them to focus on the present moment and manage their worries: ‘*you could just hear like the fire crackling and it was really calm and peaceful, so I was thinking maybe I should keep calm and try to listen to all this, and then it worked a lot*’ (Oliver).

#### Creative exploration

Many participants felt the agency to explore connected them with their creativity. This was described by Noah when participating in clay modelling, which permitted him freedom to embrace irregularity: ‘*It was really fun because you got all messy and it*’*s really fun when I get messy because it*’*s like you*’*re exploring new things and doing new things…*’. This sense of novel exploration was particularly important for participants who had missed opportunities due to their LTC. Other participants expressed how engaging in creative activities supported their self-expression:

I just think art like… shows what you like the most. And when you do it in nature, it feels a lot more fun. It’s like when you have a flat surface the bottom is always going to be nice and smooth, but I think it’s better when you have like loads of different patterns around it from the wood stool and mine had loads of bumps and holes in the bottom. It was really nice. (Rachel)

Rachel conveyed how clay modelling within nature provided a richer texture to her experiences. It appeared some participants felt controlled by their illness, having to exercise caution in daily tasks and adhere to strict to medical regimes. Creativity permitted freedom and allowed participants to momentarily let go of control. The process of exploration also extended to participant’s identity: ‘*It brought a new side to me. Like not a new side but like, it made me like try new things…*’ (Oliver). This indicated Oliver already had some sense of these aspects of himself, which were not fully realised until engaging in these experiences. Many participants shared an increased sense of courage to explore the previously unexplored.

### Theme 3: Sense of connection

This GET captured the increased sense of connection participants experienced towards their peers and towards nature. Due to experiencing shared adversity relating to illness, participants developed an innate understanding between one another. Participants also developed a sense of belonging within nature, drawing inspiration from the natural world around them.

#### A shared struggle

Participants expressed anxiety about meeting new people, prior to the WI. However, once present, all participants spoke of sharing an intuitive sense of belonging. Grace likening her experience of meeting others for the first time, to ‘*little kittens*’ coming together: ‘*So, it was like you were one of the little kittens and then you had to be introduced to all the other kittens. And we all were there. It*’*s kind of hard to explain, but we were all there*… ’. This alludes to a shared vulnerability amongst peers and comfort in overcoming isolation. The sense of belonging being ‘*hard to explain*’ was echoed by many participants, representing the difficulty in expressing this unspoken connection. Participants seemed to appreciate simply being with others who had experienced ‘*struggle*’. They emphasised shared hardship promoted empathy and understanding, despite not sharing a diagnosis: ‘*Although they might not have the same condition as me, and it might not have been as extreme. I knew they still had some condition. So, I think I felt quite nice about that. I knew I wasn*’*t left out*… ’ (Sienna). There was a sense participants drew inspiration from one another’s’ resilience and their connection facilitated an interrelating of feelings, reflecting vicariously through one another:

I saw all the other kids kind of like picking up cards and it just…. it was really interesting, and it made me think about how brave I had been. There was this kid, who picked up ‘Brave’ and it just felt nice to see other people noticing what they haven’t… it made me feel really kind of like, happy and I didn’t know… warm. (Kaylee)

Kaylee reported feeling warmed by her empathy with another group member. This had supported her to realise her own bravery in having recently undergone brain surgery to manage her epilepsy. As well as an unspoken sense of connection, it also seemed participants established a sense of connection through conversation. The WI facilitated a personal environment where participants felt validated through dialogue: ‘*You*’*re in more private places. Like one girl was telling me about her illness and all this stuff that happened to her and it was really nice to hear that…*’ (Beth). This was valued amongst participants, who often expressed they would have liked more time to make connections. Some participants, such as Noah expressed this through a desire for collectivism, rather than individualism, finding more meaning through shared activity: ‘*Maybe do more like team building activities than just doing by yourself*’.

#### Weaved into nature’s road

Additionally, all participants reported developing a connection to nature during the WI. This was symbolically described by Lydia as feeling ‘*weaved into nature*’*s* *road*’, illustrating a sense of feeling intertwined with her environment. Many participants similarly detailed developing an awareness of humans’ instinctive affinity for nature, described through a sense of belonging: ‘*I guess in nature, everything just feels like it really belongs. We would think animals belong. We would even think that people might belong there too…*’ (Summer). Summer expressed living organisms exist in their most authentic and genuine form within nature, suggesting she herself felt closely affiliated to her surroundings on the WI. This was echoed by Amara who described a sense of interconnectedness with nature: ‘*It*’*s a part of us. So basically, when we were with nature, it connects with us, as part of us. It*’*s who we are…*’

The use of ‘we’ was often expressed in the language participants used to describe their relationship to nature, emphasising participant’s embodiment of this interconnectedness and feeling part of a larger whole. This was demonstrated by the way in which participants interacted with their natural environment on the WI, seeing themselves as part of the natural landscape:

It felt like we were all connected and with nature. Like one time it rained and I think when you’re there you don’t mind getting wet as much cos you think, oh well everything is getting wet anyway so you might as well… (Beth).

Other participants, such as Rachel expressed battling with her anxieties on the WI and thoughts of leaving the day. She reported drawing inspiration from the resilience of natural life on the WI which supported her to cope:

I was just listening to it [birds chirping] and I thought, well, they’ve managed to get through with their whole life, they’ve managed, because they have predators. I was thinking, well, our life is basically a predator for us, but we can manage to live through it… and I was thinking that’s going to help me a lot…

By empathising with other living organisms, Rachel experienced a sense of equality in nature. Empathy for nature was expressed by many participants and seemed to be facilitated by active engagement with nature, through nature mindfulness and using natural resources within creative exercises. For some participants, this ignited gratitude and supported them to have a more mindful relationship with nature and a desire to preserve it:

Yeah, we have to basically think about the things, think about what we’re doing before we do it. I should feel thankful. I shouldn’t throw my food in the bin for nothing because, I could just give it away to someone who actually needs it. (Sienna)

### Theme 4: A mindful presence

This GET represented how nature engaged participant’s senses, supporting a mindful engagement to the present moment. This mindful presence aided participants to feel calmer and think more clearly, supporting them to regulate their emotions.

#### Engaging the senses

Due to experiences of anxiety, fatigue and pain, participants often described having increased sensitivity to sensory input and feeling overstimulated in their daily lives. Many reported the calm of nature felt like a distinctly different sensory experience:

In the city you hear cars, talking, shouting, like electronic noises. And in the woods, it’s just like sounds of birds, and bit of talking from the people you’re with… It’s quite nice, because you kind of have that big change of scenery that really makes you think about how different it is… (Summer)

Participants expressed that, alongside nature soothing the senses, it was also equally stimulating and promoted a mindful curiosity towards one’s surroundings. Summer expressed that she could often feel overwhelmed, and socially withdraw, due to her sensory sensitivities. The natural world provided her with sensory restoration supporting her capacity to engage in and nurture her interpersonal relationships: ‘*Well, sometimes after school if my mum asked me how was my day, I might not have wanted to say. But after [the WI], I was always like*, “*oh yeah, so first we did this*”*…*’ The sense that nature facilitated stimulation and soothing was pertinent for many participants. Some described feeling engaged in and calmed by the natural rhythms of nature. Kaylee described enjoying the sensation of the wind, which created balance and predictability: ‘*I like the feel of the flow and everything. It*’*s just really calming…*’ Amara similarly likened her experience of engaging her sense of smell during the candle making activity to ‘*floating*’, representing a sense of ease and motion within nature: ‘*It made me feel like it was kind of like floating, made me feel really calm*… ’ Alongside a feeling of airiness in nature, participants also reported a sense of earthiness and stability in nature, possibly mirroring the connection to different elements of nature: ‘*It*’*s like I don*’*t know, like grounding. I mean it*’*s* *just nice to be able to see it all around me and kind of feel it all and everything…*’ (Kaylee)

Participants’ use of language relating to their experience of ‘feeling’ within nature, indicated their immersion within their senses. It also appeared meaningful that participants were relating to their physical bodies positively, experiencing pleasure rather than discomfort related to their LTC.

#### Focusing the mind

Participants expressed that this sense of mindful presence in nature supported them to focus their thoughts and attention more purposefully within the present, rather than ruminating on the future or past. For some participants, this helped them to cope with anxious thoughts on the WI:

I might think, oh is my mum going to pick me up does she know where I am and stuff like that. And when you think of a noise, or smell, or something you can see, it kind of makes that worry go away. (Beth).

This suggested sensory engagement within nature was a useful tool to ground oneself in the present, allowing participants to gain perspective on their thoughts. This seemed to underlie Summer’s feelings, of the natural environment easing more troublesome worries of life: ‘*I feel you don*’*t really have to worry about much in nature. That*’*s more like natural worrying instead of like proper worrying…*’

Summer suggests worrying may innately be part of the human condition but can be managed better in nature. Other participants expressed being in nature changed the essence of their thinking and attention, allowing them to expand their focus and reflect on themselves and their experiences:

I feel like my attention is usually like, kind of like short attention and I can focus for a short time. But when I’m outside I feel like I can focus and everything and I look at the big picture and everything like everything’s that happened over the past few days… (Noah)

Many participants described a sense of introspection facilitated by the natural world allowing them to make sense of themselves and their experiences with clarity. Amara described similar feelings of how nature supported her to feel grounded and present with her thoughts, which allowed her to feel like a more genuine representation of herself:

It made me more down to earth. Sometimes my head is in the clouds, and I don’t concentrate, and I’m just focusing on one thing, but sometimes when I’m down to Earth, I’m like, okay, I can relax now. I can be who I am now…

This suggests participants perceived mindful presence facilitated by nature could empower them to embody a more confident and less anxious self.

## Discussion

The current study aimed to develop an understanding of how CYP with LTC and APD experience the impact of a NBTI on their wellbeing and sense of self. The study findings showed that participants perceived positive impacts of NBTIs on their mental and physical well-being and sense of self. They attributed these changes to feeling challenged, increased creativity, agency and choice, peer togetherness, belonging in nature, and sensory soothing. These findings will be discussed in relation to the study aims, research questions, and wider literature.

Previous research has explored how peer relationships and skill building in NBTIs improve freedom, creativity, empowerment and relaxation for CYP with LTC and APD. However, the current study introduced several new findings to the existing research, particularly how CYP’s engagement with the natural world, framed within the biophilia hypothesis (Wilson, 1984), highlights that nature can serve as both a therapeutic space and essential aspect of identity formation.

Disconnection from nature due to LTC-related challenges seemed to affect participants’ eco-psychological self (Barrows, 1995). A sense of belonging is crucial during identity formation (Erikson, 1968) and may be of even greater importance to CYP with LTC, who are more likely to face stigmatisation (Pittet et al., 2010). Participants in the current study expressed an innate belonging to nature. Therefore, developing a relationship with nature may provide similar benefits in alleviating loneliness and isolation as human connection (Bell-Williams et al., 2021). Participants also expressed that the environment of the WI created a sense of equality within nature, ‘*we were all connected, and with nature*’. This may have facilitated a shared human bond to the natural world, alongside participants’ shared experience of LTC, which may have deepened their sense of interpersonal connection.

Connection was an integral outcome of the WI, with participants describing feeling accepted and understood, supporting emotional wellbeing. This aligns with research illustrating the short-term benefits in social acceptance for CYP with LTC following an NBTI (Desai et al., 2014; Gillard and Allsop, 2016; Moola et al., 2014). The current study highlighted participants’ social anxiety prior to the WI and their desire for additional time to develop connections. Anxiety may pose additional challenges in engaging with peers (Loades et al., 2020). Research suggests CYP and LTC experience increased loneliness in comparison to peers (Maes et al., 2017) and peer support may improve wellbeing in NBTIs (Duerden et al., 2012). This highlights NBTIs for CYP with LTC and APD may require specific adaptation to support relationship development.

Although most studies exploring CYP with LTC’s experience of NBTIs are separated by diagnosis (Desai et al., 2014; Gillard and Allsop, 2016; Moola et al., 2014), participants in this study highlighted the importance of ‘being with’ others who experienced a ‘shared struggle’ rather than a shared condition, which was another novel finding of the study. Although it is important to understand the specific needs and experiences of different groups, the current research highlighted there may be opportunities to bring CYP with different LTC together within a supportive environment. Additionally, the study emphasised how sensory soothing and mindful presence may be important mechanisms of change within the NBTI, something that has not been thoroughly explored in previous research with LTC and APD. Mindful presence facilitated during the WI helped participants to regulate their emotions and strengthen introspection, supporting their emotional wellbeing. This supports Ulrich’s (1981) stress reduction theory and Kaplan and Kaplan’s (1989) attention restoration theory. Studies exploring NBTIs echo this, identifying increased relaxation, positive affect and decreased anxiety and depression in CYP and adults with LTC (Moola et al., 2014; Taylor et al., 2022; Trøstrup et al., 2019). Research suggests mindful presence mediates the association between nature exposure and wellbeing (Swami et al., 2019). In the current study, participants noted that mindful engagement with nature provided sensory restoration, supporting participants to manage their anxiety and connect with a more genuine sense of self. Outdoor mindfulness may be more effective than indoor mindfulness for CYP with psychological difficulties (Owens and Bunce, 2022). This suggests mindfulness outdoors in nature could be a particularly valuable tool in supporting CYP with LTC manage APD. Participants valued the invitation to listen to their needs, choose how they participate, and seek respite when overwhelmed. It is possible that being out in nature helped to facilitate a more mindful and spacious attention, allowing participants to intuitively manage anxieties, empowering their sense of self.

The study also provided findings that are supported by existing literature on NBTIs. The WI was thought to support participants to confront illness-defined self-perceptions through completing nature-based activities and developing confidence in their abilities. This is supported by literature exploring NBTIs with adults and CYP with LTC (Gillard and Allsop, 2016; Moola et al., 2014; Roberts et al., 2020; Tillmann et al., 2018; Trøstrup et al., 2019; Zhang et al., 2020). The current study also highlighted participants’ desire to challenge themselves and feel challenged by others. Moore et al. (2019) explains CYP with LTC often face constraints and limitations on activities exacerbating their condition and miss opportunities available to their peers, resulting in feelings of disempowerment. Therefore, interventions assisting CYP with LTC to push themselves out of their ‘comfort zone’ and connect with their abilities was important in developing self-esteem.

Participants expressed an enhanced sense of creativity during the WI, which supported them to explore ‘new sides’ of their identity. Barbot and Heuser (2017) suggests creativity can support CYP’s self-expression and enhance identity formation. This supports the idea NBTIs support healthy identity development and may be important for CYP with LTC experiencing APD and negative self-perceptions. Participants expressed how the WI facilitated ownership of personal space supporting their agency. This echoes research suggesting nature-based therapy fosters a more balanced power dynamic than indoor therapy (Cooley and Robertson, 2020) and allows CYP with LTC to define oneself outside of illness (Van der Riet et al., 2017). In the current study, participants expressed the importance of containment and having sensible rules, supporting the idea of ‘safe yet empowering’ environments for NBTIs (Moore et al., 2019). It is unclear from our findings whether participants had a positive affiliation with nature associated with their pre-illness identity that supported their sense of safety and ability outdoors.

Overall, the study findings extend theorised physiological, psychological and social mechanisms of NBTIs, highlighting how NBTIs may enhance traditional psychotherapeutic approaches. In contrast to traditional therapies, NBTIs may offer additional therapeutic benefits of the natural environment, supporting sensory restoration (Kaplan and Kaplan, 1989) and stress reduction (Ulrich, 1981). The sensory engagement with nature and navigating its challenges may offer a unique pathway to self-regulation and introspection, fostering emotional resilience. Additionally, NBTIs appear to facilitate a sense of agency by encouraging participants to navigate the natural environment and peer relationships independently in a safe and empowering way. This may allow participants to redefine their abilities and identity outside constraints of illness. NBTIs may support development of a holistic self, rather than improving symptoms, which differs from conventional therapeutic interventions. Further exploration into mechanisms of change, whether physiological, psychological or social, will be invaluable in understanding how NBTIs contribute to long-term wellbeing for CYP with LTC and APD.

### Strengths and limitations

This study’s qualitative design prevented inference of causal relationships or generalizable findings, but insights are potentially transferable to similar contexts. A limitation is that those who agreed to participate in the NBTI may have been more likely to have an existing biophilic relationship to nature and benefit from the intervention as opposed to those who have a biophobic relationship (Kahn, 1997). Additionally, most participants had attended more than one intervention, meaning they likely had positive experiences of the intervention. Alternatively, participants may have been more likely to discuss experiences positively, due to social desirability bias. Recruitment was mainly communicated through parents/carers and there may have been CYP wanting to be included whose views were not represented.

Participants seemed drawn to talking about joyful experiences of the NBTI, at times refraining from situating their experiences within their personal health-related experiences. The therapeutic relationship is key in developing trust with CYP (Green, 2006) and participants may have found it difficult to discuss sensitive experiences with someone they had not met previously. Additionally, CYP with LTC may be more susceptible to fatigue, impacting interview length and the affordance of time for rapport building.

### Clinical and research implications

The findings suggest short-term NBTIs may offer potential benefits to children with LTC and APD, including fostering confidence, peer and nature connection and emotion regulation. The study results indicate NBTIs may address some of the barriers faced in traditional therapeutic settings, by promoting agency and freedom enabling a more balanced power dynamic (Cooley et al., 2020). This suggests NBTIs could be a complementary approach to existing therapies for children with health vulnerabilities. Further research will need to explore how CYP and staff perceive the accessibility of this type of therapy versus traditional therapies.

It is important to acknowledge that the findings relate to a one-day NBTI formed of specific mindfulness and forest school activities and are, therefore, not transferable to all other NBTIs. Therefore, future studies should explore how the different aspects of NBTIs, such as content, duration, approach, group influence, contribute to positive outcomes. Rigorous methodologies such as randomised control trials (RCTs) are needed to establish the mechanisms of different types of NBTIs that support the wellbeing of CYP with LTC and APD and under what conditions.

While the intervention appeared to foster a sense of connection with both peers and nature, it was unclear whether participants had a pre-existing positive affiliation with nature that contributed to their sense of belonging. Further research could provide insight into the relationship between nature affiliation and wellbeing in children with LTC and APD. Moreover, participants with anxiety highlighted the importance of relationship development within the NBTI. Comparative research exploring differing approaches to group dynamics within NBTIs could provide insight into how this impacts the experiences of CYP with anxiety or other psychological difficulties. Furthermore, the current study focussed on younger children up to 13 years of age. Future research could examine adolescents’ experience of NBTIs to aid understanding of possible developmental variations in responses to NBTIs.

## Conclusion

This study contributed to the exploration of the impact of NBTIs on CYP with LTC and APD wellbeing. It highlighted the perceived positive impact on emotional, physical wellbeing and sense of self that CYP with LTC and APD derived from being on the WI. While the findings support existing literature on the benefits of NBTIs, they offer new insights into the specific aspects of the intervention that may facilitate these outcomes. Further research is needed to confirm the mechanisms within the NBTIs and the natural environment that facilitate positive change. Rigorous methodologies, such as case-series and RCTs, are needed to support the evidence base for NBTIs with CYP and APD, enabling their broader implementation across paediatric settings. Understanding their mechanisms of change will help to maximise the potential of NBTIs to support the physical and emotional wellbeing of CYP with LTC and APD.
